# *RET* Gene Alterations in Clinical Practice: A Comprehensive Review and Database Update

**DOI:** 10.3390/genes16121472

**Published:** 2025-12-09

**Authors:** Claudio Ricciardi Tenore, Eugenia Tulli, Alessia Perrucci, Roberto Bertozzi, Ludovica Fortuna, Giulia Maneri, Concetta Santonocito, Andrea Urbani, Maria De Bonis, Angelo Minucci

**Affiliations:** 1 Departmental Unit of Molecular and Genomic Diagnostics, Fondazione Policlinico Gemelli IRCCS, 00168 Rome, Italyangelo.minucci@policlinicogemelli.it (A.M.); 2 Genomics Core Facility, Gemelli Science and Technology Park (G-STeP), Fondazione Policlinico Universitario Agostino Gemelli IRCCS, 00168 Rome, Italy; 3 Department of Basic Biotechnological Sciences, Intensivological and Perioperative Clinics, Università Cattolica del Sacro Cuore, 00168 Rome, Italy; andrea.urbani@policlinicogemelli.it; 4 Departmental Unit of Chemistry, Biochemistry and Clinical Molecular Biology, Department of Diagnostic and Laboratory Medicine, Fondazione Policlinico Universitario Agostino Gemelli IRCCS, 00168 Rome, Italy

**Keywords:** *RET* gene, molecular mechanisms, *RET*-associated disorders, genetic variants, targeted therapy, *RET* mutation database, *RET* molecular analysis approach

## Abstract

**Background/Objectives:** The *RET* (Rearranged during Transfection) gene encodes a receptor tyrosine kinase. *RET* plays a critical role in embryonic development and postnatal physiology. This review provides a comprehensive overview of *RET*-associated disorders, focusing on the molecular mechanisms of *RET* activation, associated clinical phenotypes and therapeutic implications. In addition, we present an updated *RET* mutation database. **Methods:** *RET* mutation database is built through the integration and curation of data from two major *RET* mutation repositories: the Leiden Open Variation Database (LOVD) and the Cancer Knowledge Base (CKB) as well as information derived from the ClinVar database. **Results:** To date, 78 pathogenic *RET* mutations have been identified, among these, 71 (91.0%) are single nucleotide substitutions (missense variants), 2 (2.6%) are deletions, 1 (1.3%) are indels, 2 (2.6%) are nonsense mutations and 1 (1.3%) mutation affecting the introns. A pronounced clustering was observed in exons 10–11, accounting for ~60% of cases, suggesting a potential mutational hotspot with structural or functional relevance. **Conclusions**: Aberrant *RET* activation, resulting from activating missense variants, gene fusions, or overexpression, underlies a wide spectrum of human diseases. These include multiple endocrine neoplasia type 2A (MEN2A), medullary thyroid carcinoma (MTC), Hirschsprung disease, and pheochromocytoma. The existence and use of a database classifying variants in the *RET* gene plays a fundamental role in molecular diagnostics and personalized medicine.

## 1. Introduction

### 1.1. RET Structure and Function

*RET* (rearranged during transfection) is a gene that encodes a receptor tyrosine kinase (RTK). Its sequence was first identified in 1985 as being rearranged in the 3T3 fibroblast cell line following transfection with DNA from lymphoma cells. Located on chromosome 10q11.2, *RET* spans approximately 60 kilobases (kb) and is organized into 21 exons [1]. The *RET* protein acts as a receptor for glial cell-derived neurotrophic factor (GDNF) family ligands (GFLs), including GDNF, neurturin (NRTN), persephin (PSPN) and artemin (ARTN) [2].

**Figure 1:**
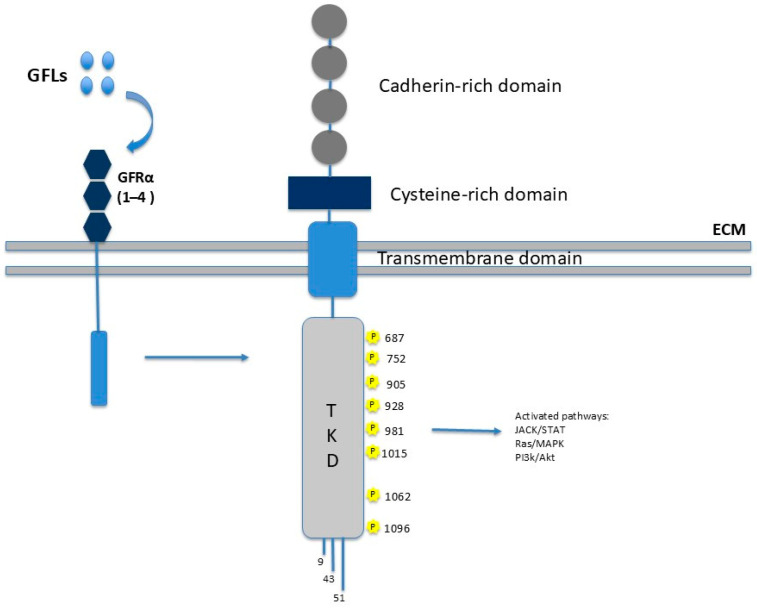
*RET* structure and function. The image depicts the main protein domains: the extracellular portion consists of the cadherin-like repeat (CLR), which mediates interactions with GFRα receptors upon his binding to GFLs. The transmembrane portion is involved in calcium binding. The intracellular portion comprises two tyrosine kinase domains (TKDs) responsible for signal transduction.

As illustrated in Figure 1, *RET* is structurally a single-pass transmembrane glycoprotein composed of three major functional domains: an extracellular ligand-binding domain; a transmembrane domain; and an intracellular tyrosine kinase domain (TKD) [3].

The hydrophobic transmembrane domain consists of amino acids 636–657 and the extracellular domain is involved in calcium binding [4].

The C-terminal intracellular domain comprises the TKD, which is interrupted by a 14-amino acid linker. This domain contains 16–18 tyrosine residues that act as phosphorylation sites for downstream adapter proteins and are critical for propagating intracellular signaling cascades [5].

The N-terminal extracellular domain of *RET* is characterized by the presence of four cadherin-like repeats (CLRs), which are involved in calcium-dependent adhesion and co-receptor interactions. This region also contains nine putative N-linked glycosylation sites that may contribute to the correct folding of proteins and the stability of receptors. Furthermore, a cysteine-rich juxta-membrane region plays a pivotal role in determining the tertiary structure of the receptor and regulating ligand-mediated dimerization [5].

*RET* is primarily expressed in neural tissues, such as the brain and autonomic nervous system (including the enteric, sympathetic, and parasympathetic nervous systems), as well as in neuroendocrine cells, including thyroid C cells, adrenal medullary cells, and parathyroid cells. It is also expressed in the developing kidney. *RET* is also expressed in the lungs and digestive system [2].

Unlike most other receptor tyrosine kinases, *RET* has a distinctive activation mechanism. Its ligands do not bind directly to the receptor but instead initially interact with GDNF family receptor α (GFRα) co-receptors. These co-receptors are glycosylated proteins that are anchored to the cell membrane via a GPI domain. The ligand–GFRα complex then recruits *RET*, promoting its homodimerization. This process induces the autophosphorylation of specific tyrosine residues in the intracellular domain, thereby triggering a cascade of intracellular signaling pathways that regulate processes such as proliferation, differentiation, migration, survival, and neuronal plasticity. *RET* activates several signaling pathways, including the RAS–RAF–MEK–ERK (MAPK) pathway, which is essential for cellular proliferation and differentiation; the PI3K–AKT–mTOR pathway, critical for survival, growth, and metabolic regulation; the JAK–STAT pathway, involved in cytokine signal transduction and immune responses; and the PKC and PKA pathways, which contribute to context-dependent cellular responses. This versatility enables *RET* to play a central role in embryonic development, particularly in the nervous system and neural crest-derived cells, as well as in adult tissue homeostasis [2].

### 1.2. RET Alterations

#### 1.2.1. In-Frame Gene Fusions Involving *RET*

*RET* fusions are somatic rearrangements that juxtapose 5′ dimerization domains from partner genes with the *RET* 3′ tyrosine kinase domain via inversion or translocation, generating oncogenic *RET* proteins [6]. *RET* fusions are recurrent somatic alterations in 6–20% of differentiated thyroid cancer (DTC) of papillary histology and occur in a smaller percentage (1–2%) of lung adenocarcinoma [5]. These fusions lead to ligand- and co-receptor-independent activation of the *RET* kinase domain (TKD) [5], triggering downstream *RAS/MAPK*, *PI3K/AKT*, and *JAK/STAT* signaling that drives proliferation and tumor progression [3,5,7,8].

In cancer, RTK fusions adopt two architectures: in 3′-kinase fusions, a partner’s N-terminus joins the RTK’s C-terminus, preserving its catalytic domain; in 5′-kinase fusions, the RTK’s N-terminal kinase domain is fused to a partner’s C-terminus [9]. Retention of an intact kinase domain is crucial for oncogenic activity [9]. Although 3′ kinase fusions predominate, 5′ kinase events, such as *FGFR2/3* and *RET* 5′ fusions, also occur, e.g., in pheochromocytoma [10].

Breakpoints typically occur within introns, maintaining an in-frame open reading frame after splicing. Most *RET* fusions retain exons 12–terminus following a breakpoint in intron 11, though alternative breakpoints may involve exons 3, 7, 9 (extracellular domain), 10 (transmembrane segment), or 11 [11]. *RET* fusions activate canonical MAPK and *PI3K–AKT* pathways [12], with the main variants being *CCDC6–RET*, *NCOA4–RET*, and *KIF5B–RET*. Oncogenic activation mechanisms include promoter swapping, which increases *RET* expression [13]; partner-mediated dimerization via coiled-coil motifs [14]; and loss of autoinhibitory segments or conformational changes enhancing kinase activity [15]. Loss of *RET*’s signal peptide and transmembrane regions can cause mislocalization and altered kinase–substrate interactions [11].

In papillary thyroid carcinoma (PTC), *CCDC6–RET* (*RET/PTC1*) and *NCOA4–RET* (*RET/PTC3*) account for ~90% of *RET*-positive cases, with breakpoints in *RET* intron 11, CCDC6 intron 1, and *NCOA4* intron 8 [11]. NCOA4–*RET* is enriched in radiation-associated cases, whereas *CCDC6–RET* is typical of sporadic disease [16]. *RET* fusions are rare in follicular thyroid carcinoma (FTC) [17].

In lung adenocarcinoma (LADC), *RET* fusions occur in ~0.35–0.88% of cases [18]. The principal *RET* fusion partners in non–small-cell lung cancer (NSCLC) are kinesin family member 5B (KIF5B) (70–90%) and *CCDC6* (10–25%), followed by *NCOA4-RET*, *TRIM33-RET*, *ZNF477P-RET*, *ERCC1-RET*, *HTR4-RET*, *CLIP1-RET* fusions (18%) (Table 1) [19]. Breakpoints within KIF5B span multiple introns, most commonly intron 15 [20].

Across tumors, *RET* fusions appear in ~0.7% of cases, including breast, colon, esophageal, ovarian, prostate, and gastric carcinomas, acute myeloid leukemia, anaplastic ganglioglioma, and Erdheim–Chester disease [18]. Another study found 0.6% of non-thyroid, non-lung malignancies positive for *RET* fusions, notably ovarian and salivary gland carcinomas [11].

Pediatric spindle mesenchymal tumors, such as infantile fibrosarcoma, may harbor *RET* fusions with partners like *MYH10*, *CLIP2*, *KIAA1217*, *SPECC1L*, *KHDRBS1*, *VCL*, and *TFG* [21]. In breast cancer, *RET* alterations occur in ~1.2% of cases, including CCDC6–*RET*, *NCOA4–RET*, *RASGEF1A–RET*, and *ZNF485–RET* fusions, or tandem duplications of exons 12–19 [22].

*RET* fusions are found in <1% of colorectal carcinomas, mainly *CCDC6–RET* and NCOA4–*RET*, with rarer partners such as *TNIP1*, *SNRNP70*, *GEMIN5*, and *RRBP1* [23]. In salivary gland secretory carcinomas lacking the typical *ETV6–NTRK3* fusion, *ETV6–RET* and *NCOA4–RET* have been identified [24].

**Table 1:** Summary of some of the main fusions involving the *RET* gene.

Neoplasm	Fusion Partner	Breakpoints (Exon/Intron)	Prevalence	Clinical Relevance	Targeted Therapy	References
Non-small cell lung cancer (NSCLC)	*KIF5B*, *CCDC6*, *NCOA4*, *PLCE1*	Intron 11 (87%), intron 10 (5%) and exon 11 (4,8%)	1–3% of NSCLC	*RET* fusions act as oncogenic drivers and define a targetable subgroup.	Selpercatinib or pralsetinib as preferred targeted agents.	[19]
Papillary Thyroid carcinoma (PTC)	*CCDC6*, *NCOA4*, *KIF5B*, *RET/PTC1*, *RET/PTC3*	Intron 11 (87%), intron 10 (5%) and exon 11 (4,8%)	10–20% of PTC	*RET*/PTC fusions drive MAPK activation and are common PTC alterations.	Selpercatinib or pralsetinib; lenvatinib or sorafenib if needed.	[11,16,17]
Breast cancer	*NCOA4*, *RASGEF1A*	Intron 11 with NCOA4 and intron 9 with RASGEF1A	1.2% of 9.693 cases	Rare events that may function as oncogenic drivers.	Selpercatinib or pralsetinib in advanced *RET*-fusion disease.	[18]
Chronic myelomonocytic leukemia (CMML)	*BCR-RET*, *FGFR1OP-RET*	Exon 12 of *RET* with fusion partners containing homodimerization domains	Rare	*RET* fusions may contribute to aggressive disease biology.	Selpercatinib; additional therapies based on disease phase.	[18]
Metastatic colorectal carcinoma (CRC)	*CCDC6-RET*, *NCOA4-RET*	Not specified	0.2% of CRC cases	Very rare but clinically actionable driver alterations.	Selpercatinib or pralsetinib; immunotherapy if MSI-H.	[23]

#### 1.2.2. Activating Missense Variants

The primary mechanism of *RET* oncogenic activation involves germline or somatic missense variants that activate the TKD of the *RET* receptor constitutively [3]. These mutations can occur in different domains of *RET*, producing distinct molecular consequences [2]:

- Amino acid substitutions in the extracellular domain, particularly at cysteine residues, promote aberrant disulfide bond formation and constitutive dimerization;
- Mutations in the transmembrane domain facilitate non-covalent dimerization of *RET* monomers;
- Mutations in the intracellular kinase domain enhance ATP binding affinity, leading to increased catalytic activity.

#### 1.2.3. Dysregulated Overexpression of *RET* Transcripts or Protein

Dysregulated *RET* overexpression, whether at the transcript or protein level, can contribute to *RET* activation. However, this mechanism is less well characterized than missense variants or gene fusions [2]. Such overexpression may amplify *RET* signaling and enhance oncogenic transformation, particularly when combined with other *RET*-activating events.

*RET* overexpression can occur through several mechanisms [25]:

- Gene amplification: In certain cancers, such as breast cancer, *RET* gene amplification leads to increased mRNA and protein levels, which enhances *RET* signaling pathways associated with tumor progression and metastasis.
- Transcriptional upregulation: Elevated expression of transcription factors or loss of repressors can lead to increased transcription of *RET*, resulting in higher levels of the receptor and enhanced downstream signaling.
- Post-transcriptional modifications: Alterations in mRNA stability or translation efficiency can increase *RET* protein levels without changing the number of copies of the gene.

#### 1.2.4. Gain of Function

Gain-of-function *RET* alterations, including both missense variants and in-frame gene fusions, lead to ligand-independent autophosphorylation and constitutive activation of downstream signaling pathways [3]. These changes drive uncontrolled proliferation, morphological transformation, and tumor progression, underscoring their critical role in oncogenesis [3,5]. *RET* alterations are detected in approximately 2% of human cancers overall, highlighting their importance as diagnostic biomarkers and therapeutic targets [26].

#### 1.2.5. Loss of Function

Loss-of-function *RET* mutations are primarily associated with developmental disorders rather than cancer. *RET*-mediated signaling plays a key role in orchestrating developmental processes through pathways such as MAPK/ERK and PI3K/AKT, which regulate cell survival, proliferation, and migration. Loss-of-function mutations disrupt these cascades, leading to tissue-specific defects that are contextually dependent on the temporal and spatial expression of *RET*. Consequently, the study of *RET* inactivation provides critical insights not only into the etiology of congenital disorders but also into fundamental principles of vertebrate development, positioning *RET* as a model for understanding how perturbations in receptor tyrosine kinase signaling translate into complex phenotypes [27].

Loss-of-function mutations in *RET* can occur through various mechanisms:

- Mutations in intracellular domain: Mutations within the intracellular tyrosine kinase domain, such as the p.Arg972Gly mutation, impair *RET*’s kinase activity, leading to reduced phosphorylation of downstream targets and disrupted signaling pathways [28].
- Mutation in extracellular domain: Mutations in the extracellular domain, such as p.Gln70Ter, can hinder the proper maturation and trafficking of the *RET* receptor to the cell surface, resulting in decreased receptor availability and impaired signal transduction [29].
- Splice site mutations: Alterations in splice sites can lead to aberrant splicing of *RET* mRNA, producing truncated or non-functional protein isoforms that lack essential signaling capabilities.

### 1.3. Pathological Phenotypic Expressions

#### 1.3.1. Pheochromocytoma

Pheochromocytoma is a rare neuroendocrine tumor that arises from chromaffin cells located in the adrenal medulla, which are responsible for the synthesis and secretion of catecholamines [30]. This neoplasm may be either benign or malignant and exhibits a wide spectrum of clinical behavior.

Pheochromocytomas are frequently associated with hereditary syndromes, including neurofibromatosis type 1 (NF1), Multiple Endocrine Neoplasia type 2 (MEN2), and Von Hippel–Lindau (VHL) disease [31]. Nevertheless, a substantial proportion of cases are sporadic in origin and represent a significant, yet often underdiagnosed, cause of secondary hypertension [31]. The clinical manifestations of pheochromocytoma are largely attributable to the excessive and episodic release of catecholamines, such as epinephrine, norepinephrine, and dopamine. These hormonal surges may result in a range of symptoms including paroxysmal hypertension, palpitations, headache, diaphoresis, and anxiety [32].

Although traditionally considered rare, advances in genetic screening have revealed that up to 35% of pheochromocytomas are associated with germline mutations in known susceptibility genes, including *RET*, *VHL*, *NF1*, and *SDHx* subunits, among others. Despite this, over half of pheochromocytoma cases occur sporadically, without any identifiable genetic or familial association, and their etiopathogenesis remains incompletely understood [31].

#### 1.3.2. Hirschsprung Disease

Hirschsprung disease (HSCR), also known as aganglionic megacolon, represents the main cause of intestinal obstruction, with an occurrence of 1/5000 in live births [33]; it is a congenital malformation of the hindgut characterized by the complete absence of parasympathetic intrinsic ganglion cells within both the submucosal (Meissner’s) and myenteric (Auerbach’s) plexuses of the distal intestinal tract [34]. This aganglionosis results in a functional obstruction due to the inability of the affected bowel segment to undergo coordinated peristaltic activity [33].

HSCR pathogenesis is attributed to a premature arrest in the craniocaudal migration of vagal neural crest-derived cells (ENCDCs) into the hindgut during embryogenesis, typically between the fifth and twelfth weeks of gestation [35]. This failure to colonize the distal bowel with ENCDCs impairs the development of the enteric nervous system (ENS) and classifies HSCR as a neurocristopathy [36].

Isolated HSCR is considered a complex genetic disorder with non-Mendelian inheritance [33]. It exhibits low and sex-dependent penetrance, is more prevalent in males, and exhibits variable phenotypic expression, particularly with respect to the length of the aganglionic intestinal segment. These features suggest polygenic involvement with contributions from one or more susceptibility genes with low penetrance [33].

In most cases, HSCR is diagnosed in the neonatal period, typically presenting with signs of intestinal obstruction. Clinical features include [37]:

- Delayed passage of meconium (>24 h after birth);
- Abdominal distension, which may be alleviated by rectal stimulation or enemas;
- Bilious vomiting;
- Episodes of neonatal enterocolitis.

However, diagnosis may be delayed until later in infancy or even adulthood in a subset of patients, particularly those with shorter aganglionic segments. These individuals often present with severe chronic constipation, persistent abdominal distension, vomiting, and failure to thrive [33].

The classic radiological presentation of HSCR includes a markedly dilated segment of proximal colon, with a characteristic transition zone leading to a narrowed, aganglionic distal segment, commonly referred to as the “aganglionic cone” [33]. Mutations in the coding sequence of the *RET* gene are identified in approximately 50% and 15% of familial and sporadic HSCR cases, respectively.

#### 1.3.3. Sporadic Medullary Thyroid Carcinoma

*RET* missense variants are highly prevalent in medullary thyroid carcinoma (MTC), occurring in more than 90% of cases, including both sporadic and hereditary forms [5].

MTC typically manifests in individuals between 40 and 60 years of age. The prognosis of this malignancy depends on several factors, including the patient’s age at diagnosis, gender (males have a poorer prognosis), the presence of local tumor invasion, and metastatic spread. The clinical course of sporadic MTC is notably variable and unpredictable. While some patients with distant metastases may experience prolonged survival, potentially extending over several years, the disease’s overall clinical behavior remains heterogeneous, reflecting its complex pathophysiology and diverse tumor biology [38].

Treatment usually involves total thyroidectomy and cervical lymph node dissection [39].

#### 1.3.4. Hereditary MTC

##### Multiple Endocrine Neoplasia Type 2A

Multiple Endocrine Neoplasia type 2A (MEN2A) accounts for about 95% of MEN2 cases. The most common clinical manifestations are medullary thyroid carcinoma (MTC), pheochromocytoma, and primary hyperparathyroidism. Familial medullary thyroid carcinoma (FMTC), a condition also categorized under MEN2A, is characterized by the presence of a thyroid neoplasm as the sole clinical feature of the syndrome [40].

Approximately 95% of MEN2A cases exhibit localized mutations in exons 10 and 11 of the *RET* gene. Although MEN2A penetrance is nearly complete, expressivity exhibits considerable variability. Notably, the age of clinical manifestation onset is closely linked to the specific *RET* gene variant present in the affected individual [41].

##### Multiple Endocrine Neoplasia Type 2B

Multiple Endocrine Neoplasia type 2B (MEN2B) represents 5% of all MEN2 cases and is characterized by the coexistence of pheochromocytoma, ganglioneuromatosis, a marfanoid habitus, and skeletal abnormalities, with primary hyperparathyroidism being a rare feature [41]. In patients with MEN2B, MTC frequently manifests in infancy and is characterized by its extremely aggressive nature. This form of MTC tends to metastasize early, often spreading to regional lymph nodes and, in many cases, to distant sites beyond the thyroid. The rapid progression and early onset of metastasis contribute to the challenging prognosis for affected individuals [40].

Approximately half of individuals diagnosed with MEN2B develop pheochromocytoma. In addition to endocrine manifestations, these patients exhibit a distinctive physical phenotype. Characteristic features include a typical appearance and a range of ophthalmologic abnormalities, such as alacrima (inability to produce tears during infancy), thickened and everted eyelids, mild ptosis, and prominently visible corneal nerves [40].

Skeletal anomalies are also prominent and often reflect a marfanoid body habitus. These include a narrow, elongated facial structure, pes cavus, pectus excavatum, high-arched palate, scoliosis, and proximal femoral epiphysiolysis. Another hallmark of MEN2B is the presence of diffuse ganglioneuromatosis affecting the entire aerodigestive tract [42].

Approximately 5% of MEN2B cases are attributed to the p.Ala883Phe mutation, located within exon 15 of the *RET* gene, while the majority of cases are caused by the p.Met918Thr mutation, found in exon 16 [40]. However, some studies suggest that patients with the p.Ala883Phe mutation may experience a less aggressive form of medullary thyroid carcinoma (MTC) than those with the p.Met918Thr mutation [43]. This observation suggests that specific *RET* gene mutations may affect the progression and severity of the disease, with p.Met918Thr typically linked to a more severe phenotype. Gastrointestinal manifestations are common, and most patients experience symptoms such as abdominal bloating, intermittent constipation, and diarrhea. In severe cases, some individuals require surgery due to intestinal obstruction.

Approximately 75% of MEN2B cases are sporadic, arising from de novo germline *RET* gene mutations. The remaining 25% are inherited and typically occur in families with a history of MEN2 [10].

### 1.4. Pharmacological Treatments

Although therapeutic strategies targeting protein tyrosine kinases (PTKs) have demonstrated substantial clinical benefit across various malignancies, the development of acquired resistance remains a significant challenge that limits long-term efficacy. Resistance can arise through bypass signaling mechanisms, whereby alternative pathways are activated to maintain proliferative and survival signals in the presence of kinase inhibition. Additionally, on-target resistance mutations may occur within the kinase domain itself, altering the conformation of the drug-binding site and diminishing drug affinity and efficacy. Elucidating the molecular underpinnings of these resistance mechanisms, identifying next-generation inhibitors, and facilitating their clinical translation are essential steps toward improving therapeutic durability and patient outcomes [44].

In response to these challenges, a novel class of agents known as selective *RET* inhibitors has been developed. Selpercatinib and pralsetinib, two of these agents, have shown significant clinical promise and received approval from the U.S. Food and Drug Administration (FDA) in 2020 for treating *RET*-altered cancers [2,3]. These agents are designed to specifically target *RET*-driven oncogenic signaling while sparing other kinases, thereby enhancing efficacy and tolerability. Notably, both selpercatinib and pralsetinib have demonstrated potent activity, including intracranial efficacy, in patients with *RET* fusion-positive or *RET*-mutant tumors [5].

Clinical evaluation of these agents has been conducted through basket trials, which enroll patients based on the presence of defined genomic alterations (e.g., *RET* rearrangements or mutations) rather than tumor histology. These trials have revealed that both agents yield high objective response rates in appropriately selected patient populations, underscoring the importance of molecular profiling to guide targeted therapy [5].

For patients with significant tumor burden, symptoms, and/or progressive disease, multi-tyrosine kinase inhibitors (MKIs), such as vandetanib and cabozantinib, have been approved for clinical use in the United States and Europe by regulatory authorities, such as the FDA and the European Medicines Agency (EMA), respectively. MKIs target *RET* (rearranged during transfection), as well as other receptors involved in tumor angiogenesis and cell proliferation [45].

### 1.5. Acquired Resistance

Selective *RET* inhibitors, such as selpercatinib and pralsetinib, have transformed the clinical management of *RET*-altered tumors. However, most patients treated with these drugs inevitably develop acquired resistance, resulting in disease progression [46]. The mechanisms underlying this resistance are heterogeneous and can be divided into two categories: target-dependent alterations, which directly involve the *RET* gene, and target-independent alterations, which include activation of alternative oncogenic pathways or phenotypic changes in the tumor. Current clinical guidelines, including those from the American Thyroid Association (ATA), recommend a tiered approach to *RET* testing. The initial analysis should focus on exons 10, 11, 13 through 16, and, in specific scenarios, exon 8 as well, based on ethnic or familial patterns. If no pathogenic variant is identified, but the clinical or familial context remains strongly suggestive, then comprehensive sequencing of the entire *RET* gene should be performed. Copy Number Variation (CNV) detection is increasingly recommended as a routine part of the molecular diagnostic workflow, either through Multiplex Ligation-dependent Probe Amplification (MLPA) or validated NGS-based approaches, especially in patients with unexplained MTC or those with a strong clinical suspicion of MTC despite negative sequencing results [40].

## 2. Materials and Methods

### Database

**Figure 2:**
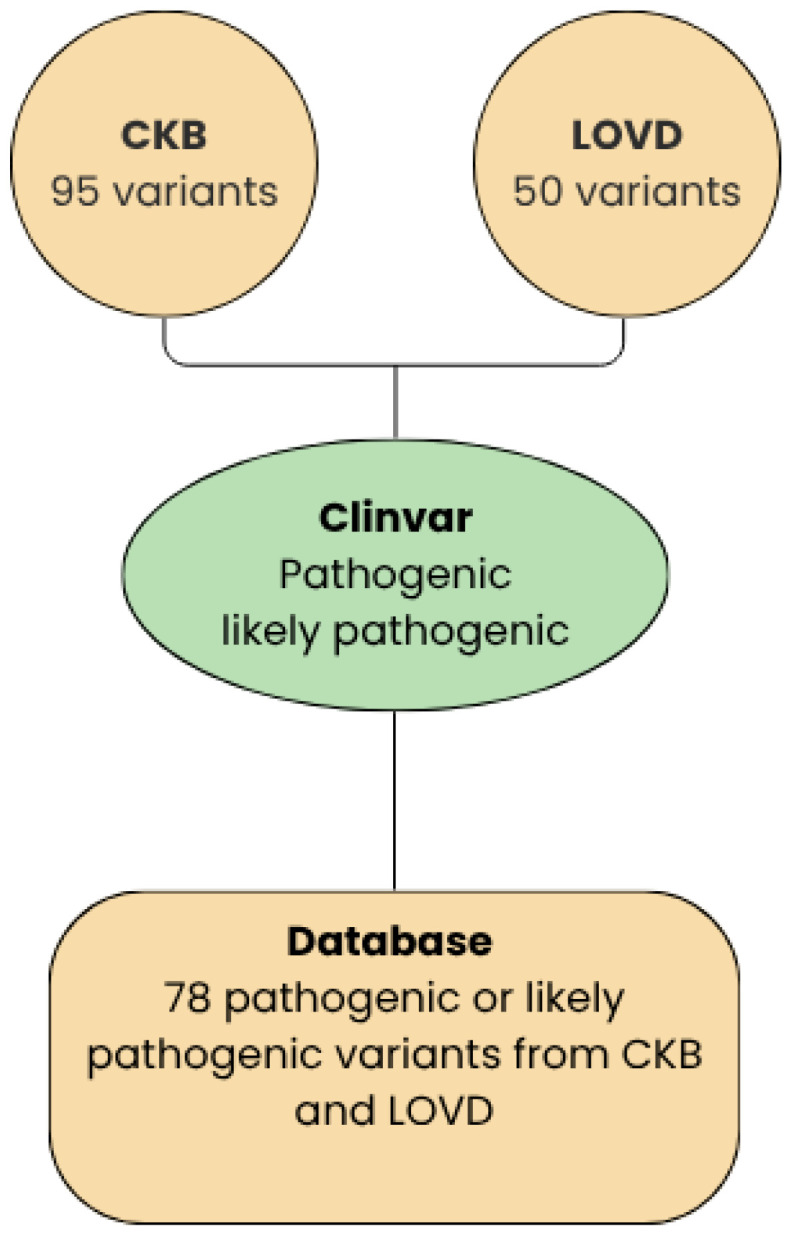
Workflow between LOVD, CKB, and ClinVar.

The RET mutation database was developed through the systematic integration and meticulous curation of data obtained from two primary repositories of RET germline variants: the Leiden Open Variation Database (LOVD) and the Cancer Knowledge Base (CKB). Both databases were last accessed in August 2025 (Figure 2). To enhance clinical interpretability, all variants collected from these sources were re-queried on ClinVar in August 2025 to distinguish, based on available evidence, between variants of uncertain significance (VUS), benign variants, and pathogenic variants. This process produced a comprehensive dataset that consolidates genomic and clinical information into a single, harmonized resource.

The primary novelty of the RET mutation database lies in the creation of a harmonized, RET-specific resource that consolidates genomic and clinical information from multiple independent repositories into a single, coherent framework. Unlike existing databases such as LOVD, ClinVar, COSMIC, or CKB—which each provide valuable but partial or heterogeneous data—this integrated database systematically unifies variant annotations, tracks discrepancies across sources, and preserves the full provenance of each entry. In doing so, it offers a level of completeness, transparency, and cross-comparability that is not currently available in any single public resource.

Construction of the database followed a structured but fully reproducible process of data extraction, normalization, cross-referencing, and manual review. RET germline variants were first collected from the Leiden Open Variation Database (LOVD) and the Cancer Knowledge Base (CKB), both accessed in August 2025. For each repository, all available genomic annotations—such as coordinates, HGVS nomenclature at the DNA and protein levels, and predicted molecular consequences—were retrieved together with any associated clinical information, including phenotype descriptions, reported hereditary syndromes, and submitter-provided comments. These data were then harmonized to a common genomic reference and the canonical RET transcript, allowing variant descriptions originating from different conventions to be reconciled. When multiple records corresponded to the same underlying variant, they were merged into a single standardized representation while preserving all source-specific annotations.

After normalization, the aggregated set of variants was compared across LOVD and CKB to identify overlapping entries as well as variants unique to one source. This cross-referencing step not only improved completeness but also exposed differences in variant coverage between repositories. To enrich the clinical interpretability of the dataset, all collected RET variants were subsequently re-queried on ClinVar, also in August 2025. For each variant, current clinical significance classifications, review status, evaluation dates, and associated conditions were retrieved. This allowed the database to distinguish, based on contemporary evidence, between pathogenic or likely pathogenic alterations, benign or likely benign variants, and variants of uncertain significance.

During this phase, particular attention was given to identifying discrepancies in pathogenicity assignments across sources. Several variants displayed conflicting interpretations—for example, being labeled as a variant of uncertain significance in one repository and as likely pathogenic in another. Rather than imposing a consensus classification, the database preserves all available interpretations, clearly indicating their sources and evidentiary bases. This approach ensures transparency and enables users to appreciate both concordant and discordant evaluations, which is essential in the context of evolving variant evidence and the ongoing reclassification of RET alterations.

Quality control steps were applied throughout the integration process. Obvious annotation errors, such as inconsistent coordinates or implausible protein changes, were flagged and either resolved through reference to original submitter notes or excluded when the underlying uncertainty could not be eliminated. All curatorial decisions—such as normalization adjustments, merging of synonymous records, and exclusion of irreconcilable entries—were documented within the database schema to maintain a complete audit trail.

The resulting resource provides a unified and traceable view of RET germline variation, combining standardized genomic descriptions with merged annotations from LOVD and CKB, enriched by up-to-date ClinVar classifications. By consolidating previously dispersed information, capturing classification discrepancies, and integrating variants that were absent from individual repositories, this database enhances the accuracy and context specificity of RET variant interpretation in hereditary syndromes and sporadic tumors. Its design not only clarifies the current landscape of RET variant knowledge but also establishes a robust foundation for monitoring future reclassification trends and supporting precision oncology efforts.

## 3. Results

### RET Pathogenic Variant Database

To date, 78 pathogenic *RET* mutations have been reported, as shown in the Supplementary Materials. Among these mutations, 71 (91.0%) are single nucleotide substitutions (missense variants), two (2.6%) are deletions, one (1.3%) is an insertion/deletion (indel), two (2.6%) are nonsense mutations, and one (1.3%) affects the introns.

**Figure 3:**
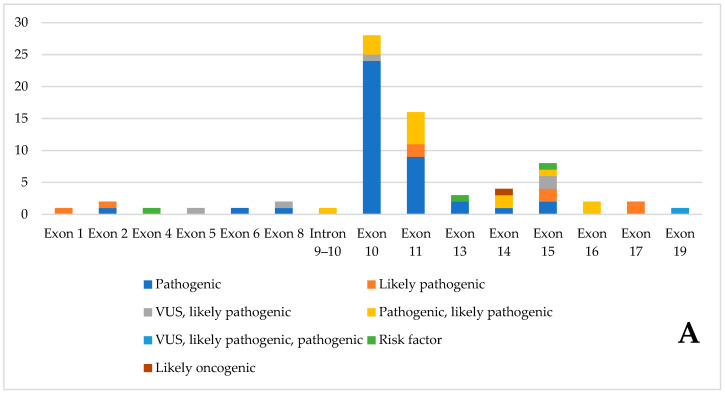

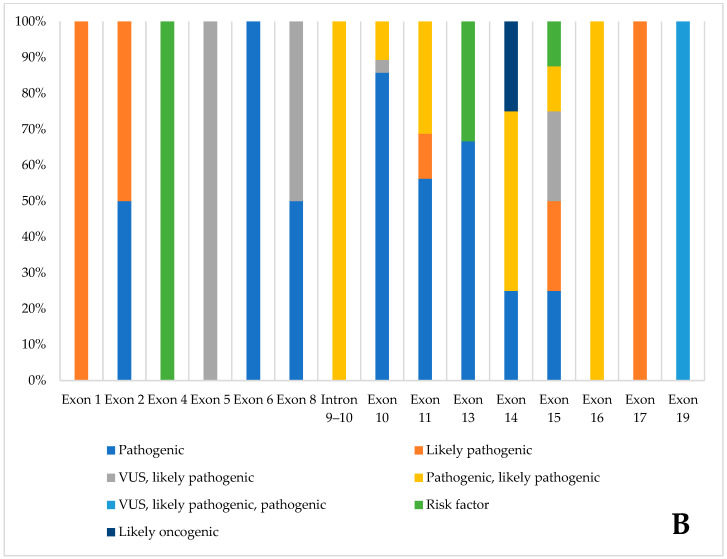
Pathogenic variants of the database, divided by exon, according to Clinvar classification. (**A**). Number of variants, divided by ClinVar classification, in the various exons (**B**). Percentage distribution of variants in individual exons.

Figure 3 summarizes the pathogenic variants found, divided by exon. Exon 10 has the highest number of pathogenic variants. Most variants are classified as pathogenic by ClinVar. Only three are risk factors, and one is likely non-oncogenic.

The table in the Supplementary Materials shows the correlation between the different molecular variants and the clinical phenotype.

The dataset reveals a clear distinction within the RET mutation spectrum between recurrent pathogenic variants, such as those affecting codons 609, 611, 618, 620, and 634, which appear multiple times across different substitution types, and rare or infrequently reported mutations. The latter include several single-entry missense or indel events distributed across exons 1, 2, 4, 5, 13, and 15. The concentration of recurrent variants in specific codons indicates the existence of functionally critical motifs, especially in the extracellular cysteine-rich domain. Disruptions in this domain are known to alter disulfide bond formation and promote ligand-independent dimerization. Conversely, rarer variants, often classified as VUS or likely pathogenic, affect residues outside these motifs and may influence secondary structural elements or regulatory regions, resulting in subtler functional consequences. Overall, the recurrence pattern highlights evolutionary and structural constraints within RET, consistent with established genotype-phenotype correlations in MEN2 and HSCR.

Analysis of the intron 9–10 region of the RET gene revealed the c.1759+1G>A variant, which is classified as pathogenic or likely pathogenic and is reported in the LOVD. Since this variant is documented in established RET mutation repositories and is not indicated as newly identified in the dataset, it confirms a previously known alteration rather than representing a novel finding [47]. Its association with unspecified “RET-related disorders” further supports the recognition of its potential impact on splicing, which is consistent with the functional relevance of intronic donor-site variants in RET-associated disease mechanisms.

## 4. Discussion

### 4.1. RET Mutation Database

Analysis of the database revealed a pronounced clustering of pathogenic variants between exons 10 and 11, which together account for approximately 60% of all identified pathogenic alterations. Although this clustering is consistent with previous reviews summarizing the genetic architecture of RET-related syndromes, the present work provides a more quantitatively robust evaluation grounded in a systematically curated, multi-source dataset. By integrating and harmonizing entries from LOVD, CKB, and ClinVar, our analysis allows direct comparison of variant frequencies across exons using standardized annotations, an analytical step that has not been performed in earlier narrative reviews. This methodology strengthens the evidence supporting exons 10 and 11 as high-impact mutational hotspots by providing updated and cross-validated proportions derived from convergent data sources. Beyond these well-recognized hotspots, the curated database also revealed potentially pathogenic variants distributed across nearly all coding exons. Our reconstruction of the full exon-by-exon pathogenicity distribution, based on harmonized variant classifications, demonstrates quantitatively that RET pathogenicity is more widespread than typically emphasized. This broader distribution provides a data-driven rationale for full-gene sequencing in cases where hotspot screening is inconclusive, expanding on the limited methodological discussion in prior reviews.

Notably, the integrated dataset also identified variants of potential relevance within introns 9 and 10. Although the contribution of non-coding regions to RET-associated disease has been sporadically hypothesized, it has not been systematically documented due to inconsistent reporting across variant repositories. By consolidating intronic entries from multiple sources and cross-checking their clinical annotations, our analysis provides the first curated overview suggesting that splice-relevant or regulatory regions may harbor functionally important alterations that warrant further investigation.

Historically, molecular analysis of RET has focused on sequencing selected hotspot exons, where pathogenic variants are most frequently found. In MEN2A and FMTC, the most commonly mutated regions are exons 10 and 11, especially codons 609, 611, 618, 620, and 634. In MEN2B, the M918T mutation is found in exon 16 in most cases, and additional variants such as A883F occur in exon 15 [48,49]. Furthermore, population studies have consistently shown that most germline RET mutations are located in these exons [50].

The broader variant distribution revealed by the integrated database—including non-hotspot exonic and intronic variants—provides analytical insights not captured in existing reviews and highlights the necessity of re-evaluating current diagnostic algorithms. By combining methodological rigor (systematic curation, cross-database reconciliation, classification harmonization) with quantitative analyses, this work extends beyond descriptive summaries and offers a refined, data-driven perspective on the mutational landscape of RET.

However, the limitations of targeted exon sequencing must be acknowledged. This approach may fail to detect rare or atypical variants located outside the canonical hotspots, including mutations in other coding exons, deep intronic regions, or regulatory elements. Additionally, the traditional focus on single nucleotide variants and small insertions/deletions excludes structural alterations, such as CNVs, which can affect *RET*. CNVs have been increasingly recognized as relevant, particularly in somatic tumor samples [51].

Another important aspect of *RET* testing is the detection of CNVs. Although relatively rare in the germline, pathogenic CNVs involving *RET* have been documented, particularly in the context of somatic alterations in medullary thyroid carcinoma (MTC). MLPA remains the gold standard for detecting exon-level deletions or duplications in *RET*. MLPA is robust, relatively simple, and well-validated for use with both germline and tumor DNA. However, its sensitivity is limited by probe coverage; it may not detect partial exon alterations or CNVs with breakpoints in non-coding regions. With the increasing use of NGS-based diagnostic panels, in silico CNV detection from read-depth data has emerged as a complementary approach. While promising, in silico CNV calls often require validation by orthogonal methods because their reliability depends on sequencing depth, coverage uniformity, and algorithm thresholds [52].

A recent comparison of MLPA and NGS-derived CNV analysis in MTC samples demonstrated sufficient concordance to support using NGS for preliminary CNV screening, provided ambiguous or borderline cases are confirmed with MLPA [53]. Interestingly, *RET* gene amplifications or deletions were observed in a subset of MTC tumors. These alterations were associated with an increased allelic frequency of *RET* driver mutations and, in some cases, a more aggressive phenotype. These findings suggest that CNV detection may offer diagnostic and prognostic value, particularly in somatic contexts.

High-throughput NGS enables the sensitive, multiplexed detection of fusions and other variants. While whole-genome approaches are costly per sample, targeted NGS panels achieve a sensitivity comparable to or better than fluorescence in situ hybridization (FISH) and are now widely implemented using diverse fusion-focused methodologies [19]. Somatic *RET* fusions are characteristic of papillary thyroid carcinoma and lung adenocarcinoma and are less common in other tumor types [11].

In thyroid cancer, the clinical relevance of *RET* fusions extends beyond targeted therapy with *RET* TKIs. In this context, molecular testing panels that include *RET* fusion detection can substantially improve diagnostic accuracy [54].

The distribution of pathogenic RET variants identified in this study is consistent with previously reported data in large-scale resources such as COSMIC and ClinVar, which identify these exons as canonical hotspots in MEN2A and FMTC cases [55,56]. Comparison with gnomAD population data confirms that most pathogenic variants detected in this cohort are either absent or extremely rare in the general population, supporting their likely pathogenic status [57]. Variants with higher allele frequencies in gnomAD are more compatible with benign polymorphisms, underlining the importance of population-based databases for variant filtering and ACMG-compliant interpretation [58].

Interestingly, potentially relevant variants were also identified in intronic and non-coding regions. Such findings emphasize that RET pathogenicity extends beyond classical hotspots. Since public databases such as COSMIC and gnomAD provide limited coverage of intronic or CNV data, complementary testing using NGS panels combined with MLPA validation remains essential for accurate molecular diagnosis [5].

Overall, these findings demonstrate that a multi-layered analytical approach—combining genomic, population, and somatic data sources—improves the reliability of RET variant interpretation, supports dynamic VUS reclassification, and informs personalized therapeutic strategies for RET-driven diseases.

### 4.2. Functional Significance of Hotspot Regions

The clustering of pathogenic RET variants within exons 10–11 and 15–16 reflects critical structural and functional domains: mutations at codons 609, 611, 618, 620, and 634 in exons 10–11 (extracellular cysteine-rich domain), disrupt normal cysteine pairing, producing aberrant intermolecular disulfide bonds. This causes ligand-independent receptor dimerization and constitutive signaling through the RAS/MAPK and PI3K pathways [6]. Variants located in exons 15–16 (tyrosine kinase domain), such as M918T alter kinase conformation, increase catalytic activity, and shift substrate specificity, resulting in more aggressive oncogenic signaling. These mutations are characteristic of MEN2B and correlate with early disease onset and aggressive MTC [40]. Understanding these structure–function relationships is essential for refining genotype-phenotype correlations and for predicting therapeutic sensitivity to RET inhibitors.

### 4.3. Implications for Precision Oncology and Genetic Counseling

RET mutations have profound implications for clinical management, influencing both therapeutic strategies and genetic counseling practices. Specific RET variants play a central role in risk stratification, as genotype–phenotype correlations guide decisions regarding the optimal timing of prophylactic thyroidectomy in children and the intensity of surveillance protocols throughout life [40,55]. In parallel, the identification of RET fusions or activating mutations has reshaped therapeutic decision-making in oncology: patients with advanced or metastatic RET-altered tumors may benefit substantially from selective RET inhibitors such as selpercatinib and pralsetinib, which have demonstrated superior efficacy and tolerability compared with older multi-kinase agents [59].

The detection of a pathogenic RET variant also has significant consequences for family members. Because these variants show strong heritability and predictable clinical behavior, cascade genetic testing in first-degree relatives is essential to identify individuals at risk and to initiate appropriate preventive or surveillance measures [55]. Furthermore, germline RET testing is recommended even in patients who appear to have sporadic medullary thyroid carcinoma, as occult germline mutations are found in 1–7% of such cases and may modify both patient management and familial screening [40].

### 4.4. Gaps in Current Testing Algorithms & Proposed Updates to ATA/NCCN Guidelines

Although substantial progress has been made in the molecular characterization of RET, several gaps remain in current diagnostic algorithms. A major limitation is the persistent reliance on hotspot-focused testing, which, while efficient, risks missing rare but clinically significant pathogenic variants located outside exons 10–11 and 15–16 [55]. This underscores the need to shift toward full-gene RET sequencing using NGS as a first-line approach, ensuring more comprehensive variant detection. Another shortcoming is the limited guidance on the identification of structural variants. Structural alterations such as copy-number changes may be overlooked without dedicated methods, and integrating MLPA or validated NGS-based CNV analysis in cases with negative sequencing results but strong clinical suspicion would improve diagnostic accuracy [52,60]

Somatic RET testing also remains insufficiently incorporated in the routine management of medullary thyroid carcinoma, despite its value in guiding the use of selective RET inhibitors and improving therapeutic decision-making [61]. Additionally, current guidelines provide little direction for evaluating deep intronic or regulatory variants, which may exert pathogenic effects through splicing disruption or transcriptional dysregulation. Expanding the use of RNA-based approaches to assess aberrant splicing could help address this important gap [62]. Collectively, these updates would modernize ATA and NCCN recommendations, aligning them more closely with contemporary genomic technologies and clinical needs.

### 4.5. Emerging RET Inhibitors

In recent years, considerable effort has been devoted to developing next-generation RET inhibitors capable of overcoming resistance mechanisms that limit the durability of response to first-generation agents. Resistance often arises through solvent-front substitutions, such as G810C, G810S, or G810R, which impair drug binding and reduce therapeutic efficacy. To address this challenge, several novel molecules are progressing through preclinical and early clinical development. One such compound is TPX-0046, a compact macrocyclic inhibitor targeting RET and SRC, specifically engineered to retain activity against solvent-front mutants [63]. Another promising candidate is BOS172738, a potent and selective RET inhibitor that has demonstrated encouraging antitumor activity in preclinical studies [62]. More recently, LOXO-260 has been introduced as a next-generation agent designed to suppress RET alterations that emerge during treatment with selpercatinib or pralsetinib, thereby offering a potential therapeutic option for patients with acquired resistance [64].

Together, these emerging inhibitors represent the next wave of precision therapeutics for RET-driven malignancies and have the potential to transform outcomes for patients who develop resistance to current standard-of-care treatments.

## 5. Conclusions

*RET* (Rearranged during transfection) is a gene that encodes a tyrosine kinase receptor (RTK) that plays a crucial role in embryonic development and postnatal physiology. Abnormal *RET* activation resulting from missense variants, gene fusions, or overexpression has been associated with various human diseases, including medullary thyroid carcinoma (MTC), Hirschsprung’s disease, and pheochromocytoma. Pharmacological strategies that target *RET* alterations have substantially advanced the therapeutic landscape of *RET*-driven malignancies, particularly through the development of selective *RET* inhibitors, such as selpercatinib and pralsetinib. These agents demonstrate robust efficacy and intracranial activity and are a significant improvement over earlier multi-kinase inhibitors because they offer enhanced selectivity and tolerability. However, clinical benefits are limited by the nearly inevitable emergence of acquired resistance mediated by target-dependent and target-independent mechanisms.

The curated *RET* mutation database contains a total of 78 pathogenic *RET* mutations and was developed through the integration of data from the Leiden Open Variation Database (LOVD), the Cancer Knowledge Base (CKB), and Supplementary information from ClinVar. Exon 10 has the highest concentration of pathogenic variants, with 28 identified. According to ClinVar, most of these variants are classified as pathogenic; only three are considered risk factors, and one is designated likely oncogenic. Analysis of the database reveals a pronounced clustering of pathogenic variants between exons 10 and 11, suggesting the presence of a hotspot in this region. However, pathogenic variants are also dispersed throughout the remaining exons, suggesting that the overall functional integrity of each exon is critical for proper *RET* activity. This widespread distribution underscores the importance of evaluating entire exons during genetic screening and functional studies. Variants located outside the primary clustered regions can still significantly contribute to disease pathogenesis.

The most suitable molecular strategy for *RET* gene analysis depends on the clinical context and available resources. A rational, stepwise approach begins with hotspot exon sequencing and simultaneous copy number variation (CNV) detection. Full-gene sequencing should be reserved for patients with negative initial results but persistent clinical suspicion. MLPA remains essential for accurately identifying CNVs, though validated NGS-based algorithms may serve as effective preliminary tools. Integrating sequencing and structural variant analysis provides the most comprehensive molecular assessment of *RET*, ensuring accurate diagnosis, effective familial screening, and optimized patient management.

The curated RET mutation database represents a novel, RET-dedicated resource that consolidates, for the first time, genomic and clinical information from multiple independent repositories into a single harmonized dataset. Unlike existing platforms, such as LOVD, ClinVar, COSMIC, or CKB, which provide valuable yet fragmented or heterogeneous information, this integrated database unifies variant annotations, applies rigorous normalization to a shared genomic reference, and systematically compares pathogenicity assessments across sources. By incorporating contemporaneous ClinVar classifications and documenting both concordant and discordant interpretations, it reveals reclassification trends and uncovers pathogenic RET variants absent from individual repositories, thereby expanding the known mutational landscape and offering a level of transparency and interpretive precision not available elsewhere.

Comprehensive knowledge of the pathological mechanisms associated with RET alterations, together with an accurate and up-to-date understanding of the genetic variants that modulate physiological and disease outcomes, is essential for advancing precision therapy. The integrated database directly supports this goal by providing an audit-ready, clinically interpretable foundation that enhances variant assessment, informs therapeutic decision-making, and guides genetic screening strategies. Ultimately, this resource facilitates the development of more targeted and effective therapies, promotes earlier and more accurate diagnosis, and enables more timely intervention in disorders driven by RET alterations.

## Data Availability

The original contributions presented in the study are included in the article/Supplementary Material, further inquiries can be directed to the corresponding author.

## Supplementary Materials

The following supporting information can be downloaded at: https://www.mdpi.com/article/10.3390/genes16121472/s1. Table S1. RET Pathogenic mutation database built through the integration and curation of data from two major RET mutation repositories: the Leiden Open Variation Database (LOVD) and the Cancer Knowledge Base (CKB) as well as information derived from the ClinVar database. Hirschsprung disease (HSCR); Multiple endocrine neoplasia type 2 (MEN2); Multiple Endocrine Neoplasia type 2A (MEN2A); Multiple Endocrine Neoplasia type 2B (MEN2B); Familial medullary thyroid carcinoma (FMTC). References [65,66,67,68,69,70,71,72,73,74,75,76,77,78,79,80,81,82,83,84,85,86,87,88,89,90,91,92,93,94,95,96,97,98,99,100,101,102] are cited in the Supplementary Materials.

## References

[1] Takahashi M., Ritz J., Cooper G.M. (1985). Activation of a Novel Human Transforming Gene, Ret, by DNA Rearrangement. Cell.

[2] Zhao L., Wang N., Zhang D., Jia Y., Kong F. (2023). A Comprehensive Overview of the Relationship between RET Gene and Tumor Occurrence. Front. Oncol..

[3] Desilets A., Repetto M., Yang S.R., Sherman E.J., Drilon A. (2023). RET-Altered Cancers—A Tumor-Agnostic Review of Biology, Diagnosis and Targeted Therapy Activity. Cancers.

[4] Iwashita T., Murakarni H., Asai N., Takahashi M. (1996). Mechanism of Ret Dysfunction by Hirschsprung Mutations Affecting Its Extracellular Domain. Hum. Mol. Genet..

[5] Shabbir A., Kojadinovic A., Shafiq T., Mundi P.S. (2023). Targeting RET Alterations in Cancer: Recent Progress and Future Directions. Crit. Rev. Oncol. Hematol..

[6] Mulligan L.M. (2014). RET Revisited: Expanding the Oncogenic Portfolio. Nat. Rev. Cancer.

[7] Chiloiro S., Capoluongo E.D., Costanza F., Minucci A., Giampietro A., Infante A., Milardi D., Ricciardi Tenore C., De Bonis M., Gaudino S. (2024). The Pathogenic RET Val804Met Variant in Acromegaly: A New Clinical Phenotype?. Int. J. Mol. Sci..

[8] Lee J.Y., Kim S.Y., Jo K.H., Mo E.Y., Kim E.S., Kim H.S., Han J.H., Moon S.D. (2022). Clinical Features and Signaling Effects of RET D631Y Variant Multiple Endocrine Neoplasia Type 2 (MEN2). Korean J. Intern. Med..

[9] Kim P., Jia P., Zhao Z. (2018). Kinase Impact Assessment in the Landscape of Fusion Genes That Retain Kinase Domains: A Pan-Cancer Study. Brief. Bioinform..

[10] Estrada-Zuniga C.M., Cheng Z.M., Ethiraj P., Guo Q., Gonzalez-Cantú H., Adderley E., Lopez H., Landry B.N., Zainal A., Aronin N. (2022). A RET::GRB2 Fusion in Pheochromocytoma Defies the Classic Paradigm of RET Oncogenic Fusions. Cell Rep. Med..

[11] Santoro M., Moccia M., Federico G., Carlomagno F. (2020). RET Gene Fusions in Malignancies of the Thyroid and Other Tissues. Genes.

[12] Plaza-Menacho I., Mologni L., McDonald N.Q. (2014). Mechanisms of RET Signaling in Cancer: Current and Future Implications for Targeted Therapy. Cell. Signal..

[13] Attié-Bitach T., Abitbol M., Gérard M., Delezoide A.L., Augé J., Pelet A., Amiel J., Pachnis V., Munnich A., Lyonnet S. (1998). Expression of the RET Proto-Oncogene in Human Embryos. Am. J. Med. Genet..

[14] Monaco C., Visconti R., Barone M.V., Pierantoni G.M., Berlingieri M.T., De Lorenzo C., Mineo A., Vecchio G., Fusco A., Santoro M. (2001). The RFG Oligomerization Domain Mediates Kinase Activation and Re-Localization of the RET/PTC3 Oncoprotein to the Plasma Membrane. Oncogene.

[15] Schram A.M., Chang M.T., Jonsson P., Drilon A. (2017). Fusions in Solid Tumours: Diagnostic Strategies, Targeted Therapy, and Acquired Resistance. Nat. Rev. Clin. Oncol..

[16] Nikiforov Y.E., Rowland J.M., Bove K.E., Monforte-Munoz H., Fagin J.A. (1997). Distinct Pattern of Ret Oncogene Rearrangements in Morphological Variants of Radiation-Induced and Sporadic Thyroid Papillary Carcinomas in Children. Cancer Res..

[17] Yakushina V.D., Lerner L.V., Lavrov A.V. (2018). Gene Fusions in Thyroid Cancer. Thyroid.

[18] Kohno T., Tabata J., Nakaoku T. (2020). REToma: A Cancer Subtype with a Shared Driver Oncogene. Carcinogenesis.

[19] Li A.Y., McCusker M.G., Russo A., Scilla K.A., Gittens A., Arensmeyer K., Mehra R., Adamo V., Rolfo C. (2019). RET Fusions in Solid Tumors. Cancer Treat. Rev..

[20] Mizukami T., Shiraishi K., Shimada Y., Ogiwara H., Tsuta K., Ichikawa H., Sakamoto H., Kato M., Shibata T., Nakano T. (2014). Molecular Mechanisms Underlying Oncogenic RET Fusion in Lung Adenocarcinoma. J. Thorac. Oncol..

[21] Davis J.L., Vargas S.O., Rudzinski E.R., López Marti J.M., Janeway K., Forrest S., Winsnes K., Pinto N., Yang S.E., VanSandt M. (2020). Recurrent RET Gene Fusions in Paediatric Spindle Mesenchymal Neoplasms. Histopathology.

[22] Paratala B.S., Chung J.H., Williams C.B., Yilmazel B., Petrosky W., Williams K., Schrock A.B., Gay L.M., Lee E., Dolfi S.C. (2018). RET Rearrangements Are Actionable Alterations in Breast Cancer. Nat. Commun..

[23] Pietrantonio F., Di Nicolantonio F., Schrock A.B., Lee J., Morano F., Fucà G., Nikolinakos P., Drilon A., Hechtman J.F., Christiansen J. (2018). RET Fusions in a Small Subset of Advanced Colorectal Cancers at Risk of Being Neglected. Ann. Oncol..

[24] Guilmette J., Dias-Santagata D., Nosé V., Lennerz J.K., Sadow P.M. (2019). Novel Gene Fusions in Secretory Carcinoma of the Salivary Glands: Enlarging the ETV6 Family. Hum. Pathol..

[25] Kakati R.T., Kim H., Whitman A., Spanheimer P.M. (2023). High Expression of the RET Receptor Tyrosine Kinase and Its Ligand GDNF Identifies a High-Risk Subset of Estrogen Receptor Positive Breast Cancer. Breast Cancer Res. Treat..

[26] Thein K.Z., Velcheti V., Mooers B.H.M., Wu J., Subbiah V. (2021). Precision Therapy for RET-Altered Cancers with RET Inhibitors. Trends Cancer.

[27] Wang H., Li Q., Zhang Z., Xiao P., Li L., Jiang Q. (2019). Functional Studies on Novel RET Mutations and Their Implications for Genetic Counseling for Hirschsprung Disease. Front. Genet..

[28] Carlomagno F., De Vita G., Berlingieri M.T., De Franciscis V., Melillo R.M., Colantuoni V., Kraus M.H., Di Fiore P.P., Fusco A., Santoro M. (1996). Molecular Heterogeneity of RET Loss of Function in Hirschsprung’s Disease. EMBO J..

[29] So M.T., LeonThomas T.Y.Y., Cheng G., TangClara C.S.M., Miao X.P., Cornes B.K., Ngo D.N., Cui L., NganElly E.S.W., LuiVincent V.C.H. (2011). RET Mutational Spectrum in Hirschsprung Disease: Evaluation of 601 Chinese Patients. PLoS ONE.

[30] Shen P., Yin N., Sun L., Liu Y., Cao X. (2023). Diagnosis and Treatment of Bilateral Adrenal Pheochromocytoma with RET Gene Mutation Combined with Medullary Sponge Kidney: A Case Report. Medicine.

[31] Gupta P.K., Marwaha B. (2024). Pheochromocytoma.

[32] Lenders J.W.M., Duh Q.Y., Eisenhofer G., Gimenez-Roqueplo A.P., Grebe S.K.G., Murad M.H., Naruse M., Pacak K., Young W.F. (2014). Pheochromocytoma and Paraganglioma: An Endocrine Society Clinical Practice Guideline. J. Clin. Endocrinol. Metab..

[33] Amiel J., Lantieri F., Burzynski G., Borrego S., Pelet A., Arnold S., Miao X., Griseri P., Brooks A.S., Antinolo G. (2008). Hirschsprung Disease, Associated Syndromes and Genetics: A Review. J. Med. Genet..

[34] Whitehouse F.R., Kernohan J.W. (1948). Myenteric Plexus in Congenital Megacolon: Study of Eleven Cases. Arch. Intern. Med..

[35] Takahashi M. (2022). RET Receptor Signaling: Function in Development, Metabolic Disease, and Cancer. Proc. Japan Acad. Ser. B Phys. Biol. Sci..

[36] Doray B., Salomon R., Amiel J., Pelet A., Touraine R., Billaud M., Attié T., Bachy B., Munnich A., Lyonnet S. (1998). Mutation of the RET Ligand, Neurturin, Supports Multigenic Inheritance in Hirschsprung Disease. Hum. Mol. Genet..

[37] Martin M.J., Steele S.R., Noel J.M., Weichmann D., Azarow K.S. (2001). Total Colonic Manometry as a Guide for Surgical Management of Functional Colonic Obstruction: Preliminary Results. J. Pediatr. Surg..

[38] Santillan V.R., Master S.R., Menon G., Burns B. (2024). Medullary Thyroid Cancer.

[39] Haddad R.I., Bischoff L., Ball D., Bernet V., Blomain E., Busaidy N.L., Campbell M., Dickson P., Duh Q.Y., Ehya H. (2022). Thyroid Carcinoma, Version 2.2022 NCCN clinical practice guidelines in oncology. JNCCN J. Natl. Compr. Cancer Netw..

[40] Wells S.A., Asa S.L., Dralle H., Elisei R., Evans D.B., Gagel R.F., Lee N., MacHens A., Moley J.F., Pacini F. (2015). Revised American Thyroid Association Guidelines for the Management of Medullary Thyroid Carcinoma. Thyroid.

[41] Associazione Italiana di Oncologia Medica (2021). AIOM Linee Guida AIOM Tumori Della Tiroide.

[42] Smith V.V., Eng E., Milla P.J. (1999). Intestinal Ganglioneuromatosis and Multiple Endocrine Neoplasia Type 2B: Implications for Treatment. Gut.

[43] Jasim S., Ying A.K., Waguespack S.G., Rich T.A., Grubbs E.G., Jimenez C., Hu M.I., Cote G., Habra M.A. (2011). Multiple Endocrine Neoplasia Type 2B with a RET Proto-Oncogene A883F Mutation Displays a More Indolent Form of Medullary Thyroid Carcinoma Compared with a RET M918T Mutation. Thyroid.

[44] Subbiah V., Shen T., Terzyan S.S., Liu X., Hu X., Patel K.P., Hu M., Cabanillas M., Behrang A., Meric-Bernstam F. (2021). Structural Basis of Acquired Resistance to Selpercatinib and Pralsetinib Mediated by Non-Gatekeeper RET Mutations. Ann. Oncol. Off. J. Eur. Soc. Med. Oncol..

[45] Koehler V.F., Adam P., Fuss C.T., Jiang L., Berg E., Frank-Raue K., Raue F., Hoster E., Knösel T., Schildhaus H.U. (2022). Treatment of RET-Positive Advanced Medullary Thyroid Cancer with Multi-Tyrosine Kinase Inhibitors-A Retrospective Multi-Center Registry Analysis. Cancers.

[46] Lin J.J., Liu S.V., McCoach C.E., Zhu V.W., Tan A.C., Yoda S., Peterson J., Do A., Prutisto-Chang K., Dagogo-Jack I. (2020). Mechanisms of Resistance to Selective RET Tyrosine Kinase Inhibitors in RET Fusion-Positive Non-Small-Cell Lung Cancer. Ann. Oncol..

[47] Gui H., Schriemer D., Cheng W.W., Chauhan R.K., Antiňolo G., Berrios C., Bleda M., Brooks A.S., Brouwer R.W.W., Burns A.J. (2017). Whole Exome Sequencing Coupled with Unbiased Functional Analysis Reveals New Hirschsprung Disease Genes. Genome Biol..

[48] Elisei R., Alevizaki M., Conte-Devolx B., Frank-Raue K., Leite V., Williams G.R. (2013). 2012 European Thyroid Association Guidelines for Genetic Testing and Its Clinical Consequences in Medullary Thyroid Cancer. Eur. Thyroid J..

[49] Raue F., Frank-Raue K. (2012). Genotype-Phenotype Correlation in Multiple Endocrine Neoplasia Type 2. Clinics.

[50] Qi X.P., Zhao J.Q., Fang X.D., Lian B.J., Li F., Wang H.H., Cao Z.L., Zheng W.H., Cao J., Chen Y. (2021). Spectrum of Germline RET Variants Identified by Targeted Sequencing and Associated Multiple Endocrine Neoplasia Type 2 Susceptibility in China. BMC Cancer.

[51] Ramone T., Mulè C., Ciampi R., Bottici V., Cappagli V., Prete A., Matrone A., Piaggi P., Torregrossa L., Basolo F. (2020). RET Copy Number Alteration in Medullary Thyroid Cancer Is a Rare Event Correlated with RET Somatic Mutations and High Allelic Frequency. Genes.

[52] Singh A.K., Olsen M.F., Lavik L.A.S., Vold T., Drabløs F., Sjursen W. (2021). Detecting Copy Number Variation in next Generation Sequencing Data from Diagnostic Gene Panels. BMC Med. Genom..

[53] Lindsey S.C., Kunii I.S., Germano-Neto F., Sittoni M.Y., Camacho C.P., Valente F.O.F., Yang J.H., Signorini P.S., Delcelo R., Cerutti J.M. (2012). Extended RET Gene Analysis in Patients with Apparently Sporadic Medullary Thyroid Cancer: Clinical Benefits and Cost. Horm. Cancer.

[54] Nikiforova M.N., Mercurio S., Wald A.I., Barbi de Moura M., Callenberg K., Santana-Santos L., Gooding W.E., Yip L., Ferris R.L., Nikiforov Y.E. (2018). Analytical Performance of the ThyroSeq v3 Genomic Classifier for Cancer Diagnosis in Thyroid Nodules. Cancer.

[55] Eng C., Plitt G. (2023). Multiple Endocrine Neoplasia Type 2. GeneReviews^®^.

[56] Castinetti F., Moley J., Mulligan L., Waguespack S.G. (2018). A Comprehensive Review on MEN2B. Endocr. Relat. Cancer.

[57] Karczewski K.J., Francioli L.C., Tiao G., Cummings B.B., Alföldi J., Wang Q., Collins R.L., Laricchia K.M., Ganna A., Birnbaum D.P. (2020). The Mutational Constraint Spectrum Quantified from Variation in 141,456 Humans. Nature.

[58] Richards S., Aziz N., Bale S., Bick D., Das S., Gastier-Foster J., Grody W.W., Hegde M., Lyon E., Spector E. (2015). Standards and Guidelines for the Interpretation of Sequence Variants: A Joint Consensus Recommendation of the American College of Medical Genetics and Genomics and the Association for Molecular Pathology. Genet. Med..

[59] Wang T., Jiang W., Yang L., Li J., Sun Y., Shi J. (2025). Overcoming Resistance in RET-Altered Cancers through Rational Inhibitor Design and Combination Therapies. Bioorg. Chem..

[60] Quenez O., Cassinari K., Coutant S., Lecoquierre F., Le Guennec K., Rousseau S., Richard A.C., Vasseur S., Bouvignies E., Bou J. (2021). Detection of Copy-Number Variations from NGS Data Using Read Depth Information: A Diagnostic Performance Evaluation. Eur. J. Hum. Genet..

[61] Nikiforov Y.E. (2011). Molecular Diagnostics of Thyroid Tumors. Arch. Pathol. Lab. Med..

[62] Porcelli T., Moccia M., De Stefano M.A., Ambrosio R., Capoluongo E., Santoro M., Hadoux J., Schlumberger M., Carlomagno F., Salvatore D. (2023). D898_E901 RET Deletion Is Oncogenic, Responds to Selpercatinib, and Treatment Resistance Can Arise via RET-Independent Mechanisms. JCO Precis. Oncol..

[63] Drilon A.E., Zhai D., Rogers E., Deng W., Zhang X., Ung J., Lee D., Rodon L., Graber A., Zimmerman Z.F. (2020). The Next-Generation RET Inhibitor TPX-0046 Is Active in Drug-Resistant and Naïve RET-Driven Cancer Models. J. Clin. Oncol..

[64] Chen M.F., Repetto M., Wilhelm C., Drilon A. (2024). RET Inhibitors in RET Fusion-Positive Lung Cancers: Past, Present, and Future. Drugs.

[65] Kjær S., Hanrahan S., Totty N., McDonald N.Q. (2010). Mammal-Restricted Elements Predispose Human RET to Folding Impairment by HSCR Mutations. Nat. Struct. Mol. Biol..

[66] Lorente-Ros M., Andrés A.M., Sánchez-Galán A., Amiñoso C., García S., Lapunzina P., Solera García J. (2020). New Mutations Associated with Hirschsprung Disease. An. Pediatr..

[67] Ben Aim L., Pigny P., Castro-Vega L.J., Buffet A., Amar L., Bertherat J., Drui D., Guilhem I., Baudin E., Lussey-Lepoutre C. (2019). Targeted Next-Generation Sequencing Detects Rare Genetic Events in Pheochromocytoma and Paraganglioma. J. Med. Genet..

[68] Oriola J., Sanchez A., Paniello B., de la Bellacasa J.P., Biarnés J. (2021). A Novel Germline Variant in RET Gene Resulting in an Additional Cysteine in a Family with Familial Medullary Thyroid Carcinoma. Fam. Cancer.

[69] Wang J., Zhang B., Liu W., Zhang Y., Di X., Yang Y., Yan D. (2016). Screening of RET Gene Mutations in Chinese Patients with Medullary Thyroid Carcinoma and Their Relatives. Fam. Cancer.

[70] Martins-Costa M.C., Lindsey S.C., Cunha L.L., Carreiro-Filho F.P., Cortez A.P., Holanda M.E., de Farias J.W.M., Lima S.B., Ferreira L.A.A., Filho P.C.M. (2018). A Pioneering RET Genetic Screening Study in the State of Ceará, Brazil, Evaluating Patients with Medullary Thyroid Cancer and at-Risk Relatives: Experience with 247 Individuals. Arch. Endocrinol. Metab..

[71] Jellins T., Hill M., Prager J.D., Francom C.R., Chan C.M., Schneider K.W., Sharma A., Herrmann B.W. (2023). Pediatric Head and Neck Manifestations Associated with Multiple Endocrine Neoplasia Syndromes. Int. J. Pediatr. Otorhinolaryngol..

[72] Elisei R., Tacito A., Ramone T., Ciampi R., Bottici V., Cappagli V., Viola D., Matrone A., Lorusso L., Valerio L. (2019). Twenty-Five Years Experience on RET Genetic Screening on Hereditary MTC: An Update on The Prevalence of Germline RET Mutations. Genes.

[73] Hedayati M., Zarif Yeganeh M., Sheikholeslami S., Afsari F. (2016). Diversity of Mutations in the RET Proto-Oncogene and Its Oncogenic Mechanism in Medullary Thyroid Cancer. Crit. Rev. Clin. Lab. Sci..

[74] Maciel R.M.B., Camacho C.P., Assumpção L.V.M., Bufalo N.E., Carvalho A.L., de Carvalho G.A., Castroneves L.A., de Castro F.M., Ceolin L., Cerutti J.M. (2019). Genotype and Phenotype Landscape of MEN2 in 554 Medullary Thyroid Cancer Patients: The BrasMEN Study. Endocr. Connect..

[75] Crockett D.K., Piccolo S.R., Ridge P.G., Margraf R.L., Lyon E., Williams M.S., Mitchell J.A. (2011). Predicting Phenotypic Severity of Uncertain Gene Variants in the RET Proto-Oncogene. PLoS ONE.

[76] Virtanen V.B., Salo P.P., Cao J., Löf-Granström A., Milani L., Metspalu A., Rintala R.J., Saarenpää-Heikkilä O., Paunio T., Wester T. (2019). Noncoding RET Variants Explain the Strong Association with Hirschsprung Disease in Patients without Rare Coding Sequence Variant. Eur. J. Med. Genet..

[77] Liu Q., Tong D., Yuan W., Liu G., Yuan G., Lan W., Zhang D., Zhang J., Huang Z., Zhang Y. (2017). Different RET Gene Mutation-Induced Multiple Endocrine Neoplasia Type 2A in 3 Chinese Families. Medicine.

[78] Figueiredo A.A., Saramago A., Cavaco B.M., Simões-Pereira J., Leite V. (2023). Familial Parathyroid Tumours—Comparison of Clinical Profiles between Syndromes. J. Endocrinol. Investig..

[79] Machens A., Lorenz K., Dralle H. (2021). Kidney Malformations and Hirschsprung’s Disease in Carriers of Cysteine Mutations in Exon 10 of the RET Proto-Oncogene. Endocrine.

[80] Okamoto M., Yoshioka Y., Maeda K., Bito Y., Fukumoto T., Uesaka T., Enomoto H. (2019). Mice Conditionally Expressing RET(C618F) Mutation Display C Cell Hyperplasia and Hyperganglionosis of the Enteric Nervous System. Genesis.

[81] Holm M., Vestergaard P., Poulsen M.M., Rasmussen Å.K., Feldt-Rasmussen U., Bay M., Rolighed L., Londero S., Pedersen H.B., Hahn C.H. (2023). Primary Hyperparathyroidism in Multiple Endocrine Neoplasia Type 2A in Denmark 1930–2021: A Nationwide Population-Based Retrospective Study. Cancers.

[82] Neocleous V., Fanis P., Frangos S., Skordis N., Phylactou L.A. (2023). RET Proto-Oncogene Variants in Patients with Medullary Thyroid Carcinoma from the Mediterranean Basin: A Brief Report. Life.

[83] Lima J.V., Scalissi N.M., de Oliveira K.C., Lindsey S.C., Olivati C., Ferreira E.N., Kater C.E. (2023). Germline Genetic Variants in Pheochromocytoma/Paraganglioma: Single-Center Experience. Endocr. Oncol..

[84] Fussey J.M., Smith J.A., Cleaver R., Bowles C., Ellard S., Vaidya B., Owens M. (2021). Diagnostic RET Genetic Testing in 1,058 Index Patients: A UK Centre Perspective. Clin. Endocrinol..

[85] Carter T.C., Kay D.M., Browne M.L., Liu A., Romitti P.A., Kuehn D., Conley M.R., Caggana M., Druschel C.M., Brody L.C. (2012). Hirschsprung’s Disease and Variants in Genes That Regulate Enteric Neural Crest Cell Proliferation, Migration and Differentiation. J. Hum. Genet..

[86] Damavandi E., Vand-Rajabpour F., Javadi-Arjmand M., Tehrani M.R.M., Larijani B., Kabuli M., Ghadami M. (2021). RET Proto-Oncogene Mutational Analysis in 45 Iranian Patients Affected with Medullary Thyroid Carcinoma: Report of a New Variant. J. Thyroid Res..

[87] Barletta Carrillo C.F., Poterico Rojas J.A., Barrionuevo Cornejo C., Casavilca Zambrano S., Pinedo Cárdenas A., Quispe Santibañez I., Castro Mujica M.d.C. (2018). Cáncer Medular de Tiroides Familiar: Reporte de Un Caso y Revisión de La Literatura. Rev. Fac. Cienc. Med. Cordoba.

[88] Elston M.S., Meyer-Rochow G.Y., Holdaway I., Conaglen J.V. (2012). Patients with RET D631Y Mutations Most Commonly Present with Pheochromocytoma and Not Medullary Thyroid Carcinoma. Horm. Metab. Res..

[89] Innella G., Rossi C., Romagnoli M., Repaci A., Bianchi D., Cantarini M.E., Martorana D., Godino L., Pession A., Percesepe A. (2020). Results and Clinical Interpretation of Germline RET Analysis in a Series of Patients with Medullary Thyroid Carcinoma: The Challenge of the Variants of Uncertain Significance. Cancers.

[90] Gudernova I., Balek L., Varecha M., Kucerova J.F., Bosakova M.K., Fafilek B., Palusova V., Uldrijan S., Trantirek L., Krejci P. (2017). Inhibitor Repurposing Reveals ALK, LTK, FGFR, RET and TRK Kinases as the Targets of AZD1480. Oncotarget.

[91] Chen L., Zhang J.X., Liu D.G., Liu H.G. (2023). A Familial Case of Multiple Endocrine Neoplasia 2A: From Morphology to Genetic Alterations Penetration in Three Generations of a Family. Diagnostics.

[92] Zhao L., Yang K.Q., Fan P., Gong D.X., Zhang L., Lu Y.T., Meng X., Zhou X.L. (2022). RET c.1901G>A and Novel SLC12A3 Mutations in Familial Pheochromocytomas. Genes.

[93] Pandit R., Khadilkar K., Sarathi V., Kasaliwal R., Goroshi M., Khare S., Nair S., Raghavan V., Dalvi A., Hira P. (2016). Germline Mutations and Genotype-Phenotype Correlation in Asian Indian Patients with Pheochromocytoma and Paraganglioma. Eur. J. Endocrinol..

[94] Belanger Deloge R., Zhao X., Luna P.N., Shaw C.A., Rosenfeld J.A., Scott D.A. (2023). High Molecular Diagnostic Yields and Novel Phenotypic Expansions Involving Syndromic Anorectal Malformations. Eur. J. Hum. Genet..

[95] Larsen L.V., Mirebeau-Prunier D., Imai T., Alvarez-Escola C., Hasse-Lazar K., Censi S., Castroneves L.A., Sakurai A., Kihara M., Horiuchi K. (2020). Primary Hyperparathyroidism as First Manifestation in Multiple Endocrine Neoplasia Type 2A: An International Multicenter Study. Endocr. Connect..

[96] Rosen E.Y., Won H.H., Zheng Y., Cocco E., Selcuklu D., Gong Y., Friedman N.D., de Bruijn I., Sumer O., Bielski C.M. (2022). The Evolution of RET Inhibitor Resistance in RET-Driven Lung and Thyroid Cancers. Nat. Commun..

[97] Romeo G., Ronchetto P., Luo Y., Barone V., Seri M., Ceccherini I., Pasini B., Bocciardi R., Lerone M., Kääriäinen H. (1994). Point Mutations Affecting the Tyrosine Kinase Domain of the RET Proto-Oncogene in Hirschsprung’s Disease. Nature.

[98] Kasak L., Lillepea K., Nagirnaja L., Aston K.I., Schlegel P.N., Gonçalves J., Carvalho F., Moreno-Mendoza D., Almstrup K., Eisenberg M.L. (2022). Actionable Secondary Findings Following Exome Sequencing of 836 Non-Obstructive Azoospermia Cases and Their Value in Patient Management. Hum. Reprod..

[99] Mathiesen J.S., Kroustrup J.P., Vestergaard P., Stochholm K., Poulsen P.L., Rasmussen Å.K., Feldt-Rasmussen U., Gaustadnes M., Ørntoft T.F., Van Overeem Hansen T. (2017). Distribution of RET Mutations in Multiple Endocrine Neoplasia 2 in Denmark 1994-2014: A Nationwide Study. Thyroid.

[100] Domingo-Gallego A., Pybus M., Bullich G., Furlano M., Ejarque-Vila L., Lorente-Grandoso L., Ruiz P., Fraga G., López González M., Piñero-Fernández J.A. (2022). Clinical Utility of Genetic Testing in Early-Onset Kidney Disease: Seven Genes Are the Main Players. Nephrol. Dial. Transplant..

[101] Tanaka A., Uemura H., Morimoto C., Nishimura A., Yoshii Y., Masui T., Ota I., Kitahara T. (2022). Four Cases of Medullary Thyroid Carcinomas Associated with Multiple Endocrine Neoplasia 2B with Rearranged during Transfection Codon M918T Mutation in the Same Family. Mol. Clin. Oncol..

[102] Jiang Q., Wang Y., Li Q., Zhang Z., Xiao P., Wang H., Liu N., Wu J., Zhang F., Chakravarti A. (2019). Sequence Characterization of RET in 117 Chinese Hirschsprung Disease Families Identifies a Large Burden of De Novo and Parental Mosaic Mutations. Orphanet J. Rare Dis..

