# Supplementary material for: RET Gene Alterations in Clinical Practice: A Comprehensive Review and Database Update
Source: Genes (Basel). 2025 Dec 9;16(12):1472. doi: 10.3390/genes16121472 (PMC12732688; doi:10.3390/genes16121472)
Supplement: Supplementary file 1 [file genes-16-01472-s001.zip › genes-3934011-supplementary.pdf]

Table S1. RET Pathogenic mutation database built through the integration and curation of data from two major RET mutation repositories: the Leiden Open Variation Database (LOVD) and the Cancer Knowledge Base (CKB) as well as information derived from the ClinVar database. Hirschsprung disease (HSCR); Multiple endocrine neoplasia type 2(MEN2); Multiple Endocrine Neoplasia type 2A(MEN2A); Multiple Endocrine Neoplasia type 2B (MEN2B); Familial medullary thyroid carcinoma (FMTC).

Location	Genotype (cDNA)	Codon Change	Protein Change	Impact list	Classification	Database	MEN2 Phenotype	References
**Exon 1**	c.95 C>T	TCG-TTG	p.Ser32Leu	Missense	Likely pathogenic	LOVD	HSCR, MEN2	[65]
**Exon 2**	c.208 C>T	CAG-TAG	p.Gln70Ter	Missense	Pathogenic	Clinvar	HSCR	-
**Exon 2**	c.277G>A	CGG-CAG	p.Gly93Ser	Missense	Likely pathogenic	LOVD	HSCR	-
**Exon 4**	c.692G>A	CGC-CAG	p.Arg231His	Missense	Risk factor	LOVD	HSCR	[36]
**Exon 5**	c.989G>A	CGG-CAG	p.Arg330Gln	Missense	VUS, likely pathogenic	LOVD	HSCR, MEN2	[4]
**Exon 6**	c.1252C>T	CGA-TGA	p.Arg418Ter	Nonsense	Pathogenic	CKB	MEN2	[66]
**Exon 8**	c.1591T>C	TGT-CGT	p.Cys531Arg	Missense	VUS, likely pathogenic	CKB	MEN2	[67]
**Exon 8**	c.1597G>T	GGC-TGC	p.Gly533Cys	Missense	Pathogenic	CKB	FMTC, MEN2A	[68]
**Intron 9-10**	c.1759+1G>A	Unknown	/	Unknown	Pathogenic, likely pathogenic	LOVD	*RET* related disorder	[47]
**Exon 10**	c.1817A>G	TAT-TGT	p.Tyr606Cys	Missense	VUS, likely pathogenic	CKB	MEN2	[69]
**Exon 10**	c.1825T>C	TGC-CGC	p.Cys609Arg	Missense	Pathogenic	LOVD, CKB	FMTC, HSCR, MEN2	[50]
**Exon 10**	c.1825T>G	TGC-GGC	p.Cys609Gly	Missense	Pathogenic	LOVD, CKB	MEN2A	[70]
**Exon 10**	c.1826G>A	TGC-TAC	p.Cys609Tyr	Missense	Pathogenic	LOVD, CKB	MEN2A, HSCR FMTC	[71]
**Exon 10**	c.1826G>C	TGC-TCC	p.Cys609Ser	Missense	Pathogenic	LOVD, CKB	MEN2A	[72]

**Exon 10**	c.1826G>T	TGC-TTC	p.Cys609Phe	Missense	Pathogenic	LOVD, CKB	MEN2	[73]
**Exon 10**	c.1827C>G	TGC-TGG	p.Cys609Trp	Missense	Pathogenic	CKB	MEN2	[74]
**Exon 10**	c.1831T>A	TGC-AGC	p.Cys611Ser	Missense	Pathogenic, likely pathogenic	CKB	MEN2	[75]
**Exon 10**	c.1831T>C	TGC-CGC	p.Cys611Arg	Missense	Pathogenic, likely pathogenic	CKB	MEN2, HSCR	[76]
**Exon 10**	c.1831T>G	TGC-GGC	p.Cys611Gly	Missense	Pathogenic	CKB	Unknown	[74]
**Exon 10**	c.1832_1833delinsAT	TGC-TAT	p.Cys611Tyr	Indel	Pathogenic	LOVD	MEN2	[77]
**Exon 10**	c.1832G>A	TGC-TAC	p.Cys611Tyr	Missense	Pathogenic	LOVD, CKB	MEN2	[78]
**Exon 10**	c.1832G>T	TGC-TTC	p.Cys611Phe	Missense	Pathogenic	LOVD, CKB	MEN2A	[77]
**Exon 10**	c.1833C>G	TGC-TGG	p.Cys611Trp	Missense	Pathogenic	LOVD, CKB	MEN2A	[74]
**Exon 10**	c.1852T>A	TGC-AGC	p.Cys618Ser	Missense	Pathogenic	LOVD, CKB	MEN2A, MEN2, HSCR	[40]
**Exon 10**	c.1852T>C	TGC-CGC	p.Cys618Arg	Missense	Pathogenic	LOVD, CKB	FMTC, MEN2A, MEN2B	[72]
**Exon 10**	c.1852T>G	TGC-GGC	p.Cys618Gly	Missense	Pathogenic	LOVD, CKB	MEN2A, MEN2B, FMTC,HSCR	[55]
**Exon 10**	c.1853G>A	TGC-TAC	p.Cys618Tyr	Missense	Pathogenic	LOVD, CKB	MEN2	[50]
**Exon 10**	c.1853G>C	TGC-TCC	p.Cys618Ser	Missense	Pathogenic	LOVD	MEN2A, FMTC,MEN2	[79]
**Exon 10**	c.1853G>T	TGC-TTC	p.Cys618Phe	Missense	Pathogenic	LOVD, CKB	MEN2A	[80]

**Exon 10**	c.1858T>A	TGC-AGC	p.Cys620Ser	Missense	Pathogenic, likely pathogenic	CKB	MEN2	[40]
**Exon 10**	c.1858T>C	TGC-CGC	p.Cys620Arg	Missense	Pathogenic	LOVD, CKB	MEN2A, HSCR	[81]
**Exon 10**	c.1858T>G	TGC-GGC	p.Cys620Gly	Missense	Pathogenic	LOVD, CKB	MEN2A, HSCR	[45]
**Exon 10**	c.1859G>A	TGC-TAC	p.Cys620Tyr	Missense	Pathogenic	LOVD, CKB	MEN2A, HSCR	[82]
**Exon 10**	c.1859G>C	TGC-TCC	p.Cys620Ser	Missense	Pathogenic	LOVD	FMTC, MEN2A	[83]
**Exon 10**	c.1859G>T	TGC-TTC	p.Cys620Phe	Missense	Pathogenic	LOVD, CKB	MEN2A, MEN2B	[84]
**Exon 10**	c.1860C>G	TGC-TGG	p.Cys620Trp	Missense	Pathogenic	LOVD, CKB	MEN2A, HSCR	[40]
**Exon 10**	c.1860C>A	TGC-TGA	p.Cys620Ter	Missense	Pathogenic	LOVD	HSCR	[85]
**Exon 11**	c.1888T>C	TGC-CGC	p.Cys630Arg	Missense	Pathogenic	CKB	MEN2	[86]
**Exon 11**	c.1888T>A	TGC-AGC	p.Cys630Ser	Missense	Likely pathogenic	CKB	Hereditary cancer-predisposing syndrome	[87]
**Exon 11**	1889 G>A	TGC-TAC	p.Cys630Tyr	Missense	Pathogenic	Clinvar	MEN2A	[8]
**Exon 11**	c.1891G>T	GAC-TAC	p.Asp631Tyr	Missense	Pathogenic	LOVD, CKB	MEN2	[88]
**Exon 11**	c.1893_1898del	GACGAGCT-GAG	p.Asp631_Leu633delinsGlu	Deletion	Likely pathogenic	CKB	MEN2B	[89]
**Exon 11**	c.1900T>A	TGC-AGC	p.Cys634Ser	Missense	Pathogenic	LOVD, CKB	MEN2	[90]
**Exon 11**	c.1900T>C	TGC-CGC	p.Cys634Arg	Missense	Pathogenic, likely pathogenic	LOVD, CKB	MEN2A, MEN2B, HSCR, Pheochromocytoma	[8]

**Exon 11**	c.1900T>G	TGC-GGC	p.Cys634Gly	Missense	Pathogenic	LOVD, CKB	MEN2A, FMTC, Pheochromocytoma	[91]
**Exon 11**	c.1901G>A	TGC-TAC	p.Cys634Tyr	Missense	Pathogenic	LOVD, CKB	MEN2A, MEN2B, HSCR, Pheochromocytoma	[92]
**Exon 11**	c.1901G>C	TGC-TCC	p.Cys634Ser	Missense	Pathogenic	LOVD, CKB	MEN2A, MEN2B, Pheochromocytoma	[72]
**Exon 11**	c.1901G>T	TGC-TTC	p.Cys634Phe	Missense	Pathogenic	LOVD, CKB	MEN2A, FMTC, Pheochromocytoma	[93]
**Exon 11**	c.1902C>G	TGC-TGG	p.Cys634Trp	Missense	Pathogenic	LOVD, CKB	MEN2A, Pheochromocytoma	[8]
**Exon 11**	c.1947G>A	TCG-TCA	p.Ser649=	Missense	Pathogenic, likely pathogenic	LOVD	FMTC, MEN2A, MEN2B, HSCR,	[94]
**Exon 11**	c.1996A>G	AAG-GAG	p.Lys666Glu	Missense	Pathogenic, likely pathogenic	CKB	FMTC, HSCR, MEN2A, MEN2B	[95]
**Exon 11**	c.1998G>C	AAG-AAC	p.Lys666Asn	Missense	Pathogenic, likely pathogenic	CKB	MEN2A, MEN2B, HSCR, FMTC	[96]
**Exon 11**	c.1998G>T	AAG-AAT	p.Lys666Asn	Missense	Pathogenic, likely pathogenic	LOVD	MEN2A, FMTC, HSCR, MEN2B, Pheochromocytoma	[96]
**Exon 13**	c.2293T>C	TCC-CCC	p.Ser765Pro	Missense	Risk factor	CKB	HSCR	[97]
**Exon 13**	c.2304G>C	GAG-GAC	p.Glu768Asp	Missense	Pathogenic	LOVD, CKB	MEN2, FMTC	[84]
**Exon 13**	c.2370G>T	TTG-TTT	p.Leu790Phe	Missense	Pathogenic	LOVD, CKB	MEN2A, FMTC, HSCR	[98]
**Exon 14**	c.2410G>A	GTG-ATG	p.Val804Met	Missense	Pathogenic, likely pathogenic	LOVD, CKB	MEN2A, FMTC, HSCR	[55]

**Exon 14**	c.2410G>C	GTG-CTG	p.Val804Leu	Missense	Pathogenic, likely pathogenic	LOVD, CKB	MEN2A	[74]
**Exon 14**	c.2410G>T	GTG-TTG	p.Val804Leu	Missense	Pathogenic	Clinvar	FMTC, MEN2A	[99]
**Exon 14**	c.2428G>T	GGC-TGC	p.Gly810Cys	missense	Likely oncogenic	CKB	Neoplasm	[44]
**Exon 15**	c.2617C>T	CGG-TGG	p.Arg873Trp	Missense	Likely pathogenic	CKB	HSCR	[44]
**Exon 15**	c.2647_2648delinsTT	GCT-TTT	p.Ala883Phe	indel	Pathogenic, likely pathogenic	CKB	MEN2B	[84]
**Exon 15**	c.2656C>T	CGG-TGG	p.Arg886Trp	Missense	VUS, Likely pathogenic	CKB	MEN2, MTC	[100]
**Exon 15**	c.2671T>G	TCG-GCG	p.Ser891Ala	Missense	Pathogenic	LOVD, CKB	MEN2A,MEN2B, FMTC, HCSR	[50]
**Exon 15**	c.2672C>T	TCG-TTG	p.Ser891Leu	Missense	Likely pathogenic	CKB	MEN2A, MEN2B, FMTC, Pheochromocytoma	
**Exon 15**	c.2689C>T	CGA-TGA	p.Arg897X	Nonsense	Pathogenic	LOVD	MEN2	[85]
**Exon 15**	c.2690G>A	CGA-CAA	p.Arg897Gln	Missense	Risk factor	CKB	HSCR	[97]
**Exon 15**	c.2711C>T	TCC-TTC	p.Ser904Phe	Missense	VUS, Likely pathogenic	CKB	MEN2A	[89]
**Exon 16**	c.2752A>G	ATG-GTG	p.Met918Val	Missense	Pathogenic, likely pathogenic	CKB	MEN2A	[50]
**Exon 16**	c.2753T>C	ATG-ACG	p.Met918Thr	Missense	Pathogenic, likely pathogenic	LOVD, CKB	MEN2A, MEN2B, Pheochromocytoma, FMTC	[101]
**Exon 17**	c.2905C>T	CGG-TGG	p.Arg969Trp	Missense	Likely pathogenic	LOVD		
**Exon 17**	c.2914A>G	AGG-GGG	p.Arg972Gly	Missense	Likely pathogenic	CKB	HSCR, FMTC, MEN2A, MEN2B	[97]
**Exon 19**	c.3185A>G	TAT-TGT	p.Tyr1062Cys	Missense	VUS, likely pathogenic, pathogenic	CKB	HSCR, MEN2A, MEN2B, FMTC	[102]
